# Mindfulness-Based Cognitive Therapy Regulates Brain Connectivity in Patients With Late-Life Depression

**DOI:** 10.3389/fpsyt.2022.841461

**Published:** 2022-02-14

**Authors:** Hui Li, Wei Yan, Qianwen Wang, Lin Liu, Xiao Lin, Ximei Zhu, Sizhen Su, Wei Sun, Manqiu Sui, Yanping Bao, Lin Lu, Jiahui Deng, Xinyu Sun

**Affiliations:** ^1^Peking University Sixth Hospital, Peking University Institute of Mental Health, NHC Key Laboratory of Mental Health (Peking University), National Clinical Research Center for Mental Disorders (Peking University Sixth Hospital), Beijing, China; ^2^Beijing Xi Cheng District Pingan Hospital, Beijing, China; ^3^National Institute on Drug Dependence and Beijing Key Laboratory of Drug Dependence, Peking University, Beijing, China; ^4^Peking-Tsinghua Center for Life Sciences and PKU-IDG/McGovern Institute for Brain Research, Peking University, Beijing, China

**Keywords:** late-life depression, mindfulness-based cognitive therapy, magnetic resonance imaging, functional connection, amygdala, middle frontal gyrus

## Abstract

Late-life depression (LLD) is an important public health problem among the aging population. Recent studies found that mindfulness-based cognitive therapy (MBCT) can effectively alleviate depressive symptoms in major depressive disorder. The present study explored the clinical effect and potential neuroimaging mechanism of MBCT in the treatment of LLD. We enrolled 60 participants with LLD in an 8-week, randomized, controlled trial (ChiCTR1800017725). Patients were randomized to the treatment-as-usual (TAU) group or a MBCT+TAU group. The Hamilton Depression Scale (HAMD) and Hamilton Anxiety Scale (HAMA) were used to evaluate symptoms. Magnetic resonance imaging (MRI) was used to measure changes in resting-state functional connectivity and structural connectivity. We also measured the relationship between changes in brain connectivity and improvements in clinical symptoms. HAMD total scores in the MBCT+TAU group were significantly lower than in the TAU group after 8 weeks of treatment (*p* < 0.001) and at the end of the 3-month follow-up (*p* < 0.001). The increase in functional connections between the amygdala and middle frontal gyrus (MFG) correlated with decreases in HAMA and HAMD scores in the MBCT+TAU group. Diffusion tensor imaging analyses showed that fractional anisotropy of the MFG-amygdala significantly increased in the MBCT+TAU group after 8-week treatment compared with the TAU group. Our study suggested that MBCT improves depression and anxiety symptoms that are associated with LLD. MBCT strengthened functional and structural connections between the amygdala and MFG, and this increase in communication correlated with improvements in clinical symptoms.

Randomized Controlled Trial; Follow-Up Study; fMRI; Brain Connectivity

## Introduction

Depression that occurs after 60–65 years of age is typically referred to as late-life depression (LLD), with a lifetime prevalence of 16% (1, 2). Late-life depression is an important public health concern among the aging population, severely affecting psychological, social, and biological functions (3). According to data from the World Health Organization, LDD is currently the leading cause of disability worldwide (4). The most common treatments for LLD are antidepressants, but side effects and other limitations can hamper their efficacy. Older adults are considered a vulnerable population because they are more likely to suffer from chronic medical conditions and are sensitive to adverse effects of antidepressants (5). Risks of drug interactions and undesirable side effects increase because of metabolic abnormalities that occur with aging and multiple prescribed medications that are taken for comorbidities (5). Older patients with depression require extra care from clinical professionals because of high rates of relapse and recurrence (6). Psychotherapy can play a significant role in the treatment of psychiatric disorders. Psychotherapy helps patients with mood disorders deal with maladaptive thinking and develop a positive mindset (6, 7). The combination of psychotherapy and pharmacotherapy was shown to be an effective approach to treat the acute phase of depression and preventing relapse and recurrence (7).

Mindfulness-based cognitive therapy (MBCT) is an evidence-based psychotherapeutic intervention that integrates several essential elements of cognitive behavioral therapy with mindfulness meditation (8). Mindfulness training focuses on the progressive acquisition of mindful awareness to ameliorate stress and reduce negative feelings and thoughts. Thus, MBCT may be a good option to treatment stress-related disease. MBCT is currently recommended by several national clinical guidelines as a prophylactic treatment for recurrent major depressive disorder (9, 10), and it is considered a cost-effective intervention. MBCT usually consists of 8-week group sessions and individual daily homework between sessions. Since the first edition of the MBCT manual was published in 2002, mounting interest in MBCT and its clinical potential has been seen in treating depressive disorders (11). Previous studies demonstrated that MBCT is an effective nonpharmacological approach to reduce the risk of depression relapse/recurrence in young adults with major depression (12) and patients with LLD in primary care (13, 14). However, unknown are whether and how specific circuits are affected by MBCT.

Many studies indicate that abnormal structural and functional alterations of the brain underlie the pathophysiology of LDD (15–17). Negative correlations were found between a greater number of depressive episodes and a reduction of hippocampal volume (18), with thinning of the medial prefrontal cortex (mPFC) (19). Illness duration also correlated with a volume reduction of the hippocampus, putamen, insula, and mPFC (20–22). Resting-state functional magnetic resonance imaging (fMRI) studies reported a reduction of connectivity between the amygdala and dorsal frontal regions in LLD patients (23), with disruptions of the fronto-parietal network (24). Brain connectivity between the fronto-parietal network and regions that are involved in sensory information processing were restored after MBCT in patients with major depression (25). Additionally, meditation training is a vital part of MBCT that induces brain network changes in functional and structural connectivity, such as the fronto-parietal network, default mode network, and salience network (26, 27). Prior studies that investigated neural mechanisms of emotion regulation reported that meditation training heightened functional connectivity between the amygdala and mPFC (28). Some attempts have been made to provide initial evidence of a decrease in structural brain connectivities in MDD (29, 30). A decrease in fractional anisotropy (FA) of the white matter tract that connects the subgenual anterior cingulate cortex and amygdala was observed in adolescents with depression (30). However, an increase in radial diffusivity of the right uncinate fasciculus was also reported in patients with MDD (29). Still unclear, however, is how MBCT affects brain structure and function to improve symptoms of LDD.

The present study explored whether MBCT as an adjunctive treatment to treatment as usual (TAU) in LDD patients has superior outcomes compared with TAU alone. Brain imaging was performed to evaluate the possible mechanism of adjunctive MBCT in LDD patients by analyzing brain connectivity and potential biological correlates. We also investigated whether white matter integrity between the amygdala and MFG (i.e., two brain regions that have shown to be important in MDD) supports abnormal functional connectivity between these two brain regions (31, 32). Diffusion-weighted images were collected, and the FA of fiber tracts that connect the amygdala and MFG was compared between patients who received MBCT combined with TAU or TAU alone. Our findings may contribute to new non-pharmaceutical strategies for the treatment of elderly patients with LLD.

## Materials and Methods

### Participants

We recruited outpatients with depression who were over 60 years of age, with no upper age limit, from May 2018 to June 2019. This study was approved by the Ethics Committee of Peking University Sixth Hospital. All patients provided written informed consent to participate in the study. All of the subjects were diagnosed with depression by two experienced psychiatrists using the *Diagnostic and Statistical Manual of Mental Disorders*, 4th edition (DSM-IV), and Structured Clinical Interview for the DSM (SCID). The severity of depression was assessed using the 24-item Hamilton Depression Scale (HAMD). The severity of anxiety was assessed using the Hamilton Anxiety Scale (HAMA). Overall cognitive function was rated using the Mini-Mental State Examination (MMSE). For all of the subjects, the exclusion criteria included the following: (1) current or previous diagnosis of schizophrenia, bipolar disorder, substance abuse, substance-induced depression, or dementia, (2) MMSE score <26, (3) severe somatic disease (e.g., brain tumors, metastatic cancer, or unstable cardiac, hepatic, or renal disease), (4) treatment with antidepressants (except selective serotonin reuptake inhibitors [SSRIs] and selective serotonin-norepinephrine reuptake inhibitors [SNRIs]), (5) current suicidal ideation, (6) left handed, and (7) any contraindication to fMRI (e.g., having a pacemaker or implanted metal). In this study, participants were informed about assignment while assessors remained blind.

### Study Design

**Figure 1:**
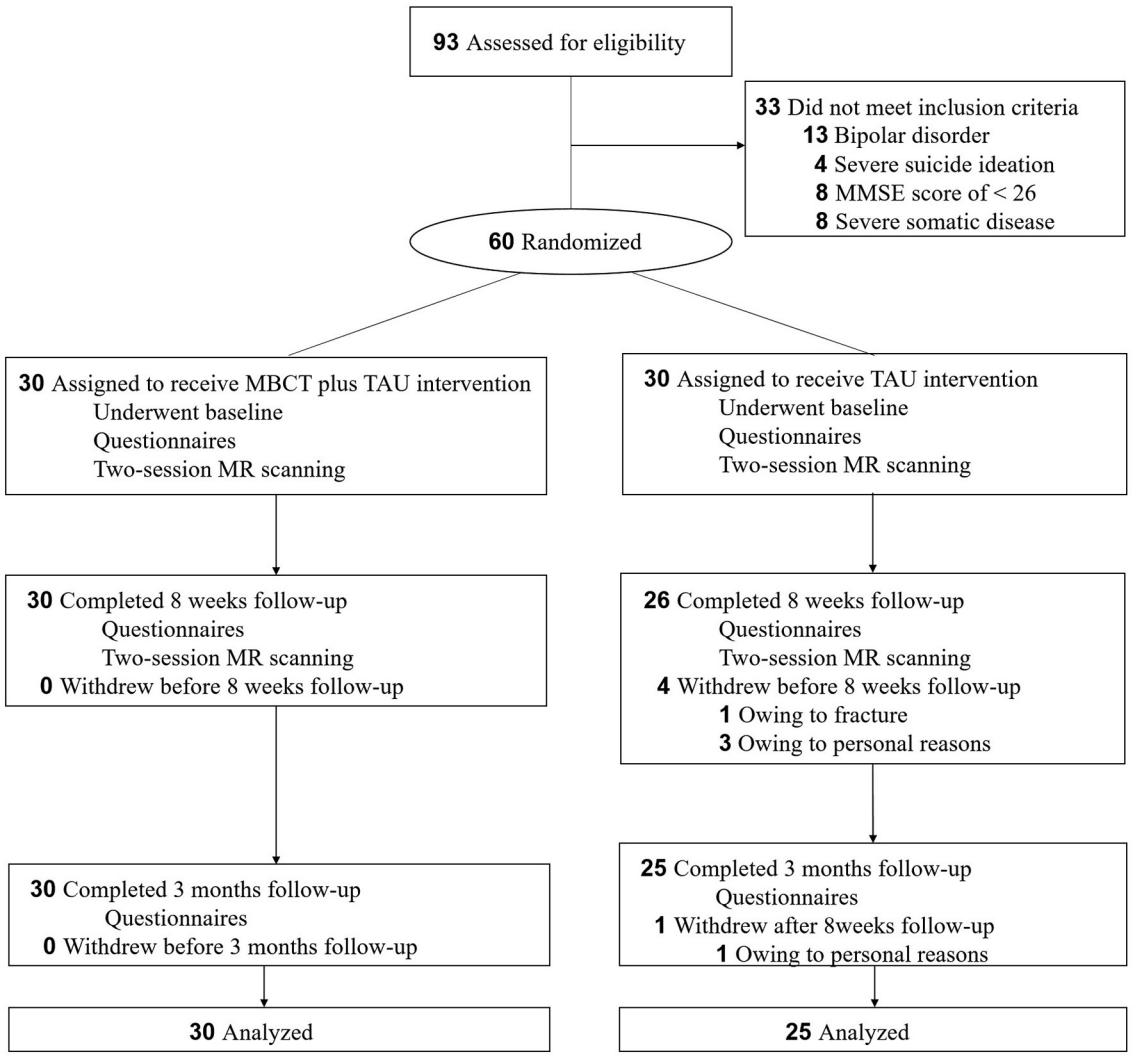
Flow chart of the study.

This was a single-center, randomized, parallel-group, controlled trial that was registered with ClinicalTrials.gov (no. ChiCTR1800017725). The study consisted of three phases: screening phase, 8-week treatment phase, and up to a 3-month follow-up phase. Following baseline measure completion, eligible patients were randomly divided into the MBCT+TAU group or TAU group. The MBCT+TAU group was given 8 weeks of the MBCT intervention with current antidepressant treatments. The TAU group continued to receive their usual antidepressant treatments only (Figure 1). In the MBCT+TAU group, 22 patients received SSRIs (15 escitalopram, 20 mg/day; 5 sertraline, 150-200 mg/day; 2 fluoxetine, 20 mg/day), and eight patients received SNRIs (5 duloxetine, 60–90 mg/day; 3 venlafaxine, 150–225 mg/day). In the TAU group, 23 patients received SSRIs (16 escitalopram, 20 mg/day; 7 sertraline, 150–200 mg/day), and seven received SNRIs (3 duloxetine, 60–90 mg/day; 4 venlafaxine, 150–225 mg/day).

The MBCT protocol was based on a manualized 8-week meditation-based skills-training group program (8) and adapted to be more suitable for elderly individuals with depression. MBCT was adapted to cater older adults' physical condition and prevent them falls. Furthermore, we would provide online support offers for them to better understanding MBCT program. During 8-week treatment, the guided meditations ranged from 60 to 90 min once weekly. Mindfulness were supervised 45 min daily by themselves. Patients were recommended to practice mindfulness voluntarily at home. The time of voluntary mindfulness practice were recorded per day by a mindfulness practice log. We visited patients in the TAU group weekly to ask about adverse events. The TAU group did not receive any other psychotherapy. We evaluated changes in depressive and anxiety symptoms using the 24-item HAMD as the primary outcome and HAMA as the secondary outcome at baseline, at the end of 8 weeks of treatment, and at the 3-month follow-up.

### Functional Magnetic Resonance Imaging

To further explore possible mechanisms of MBCT, fMRI was performed to measure changes in brain function during the resting state (33, 34) in LLD patients before and after treatment at baseline (pre-test) and 8 weeks (post-test). fMRI data for all participants were acquired using a research-dedicated 3.0 T GE EXCITE HD scanner (GE Medical Systems, Milwaukee, WI, USA) at Peking University Sixth Hospital with an 8-channel head coil. For each participant, high-resolution T1-weighted structural images were acquired using the following parameters: field of view (FOV) = 25.6 cm^3^, flip angle = 12°. Eight-min resting-state fMRI scans were performed using a gradient-echo echo-planar imaging (EPI) sequence (FOV = 22.0 cm^3^, TR = 2,000 ms, TE = 30 ms, flip angle = 90°, number of slices = 43, total scans = 240). We then performed diffusion tensor imaging (DTI) scans by a gradient-echo EPI sequence (FOV = 22.0 cm^3^, TR = 2,000 ms, TE = 30 ms, flip angle = 90°, number of slices = 43, total scans = 240). To reduce motion artifacts, the participant's head was fixed with foam pads on the scanner bed. Each participant was instructed to stay still and awake during data acquisition.

### Diffusion Tensor Imaging Data

Diffusion tensor imaging data were processed using tools that are included in the Functional MRI of the Brain (FMRIB) Software Library (FSL), which is used for motion and eddy-current correction. A diffusion tensor model was fit at each voxel using DTIFIT. For individual FA images, the FMRIB Diffusion Toolbox (FDT) was used to fit a diffusion tensor to raw diffusion data, and the brain was extracted using the Brain Extraction Tool (BET). All subjects' FA images were aligned into a common space using the FNIRT nonlinear registration tool. The threshold of the mean FA skeleton image was 0.2.

Data preprocessing and FA values for each white matter tract were calculated using automated whole-brain atlas-based tractography with above method. Preprocessing was performed using the FSL. First, non-brain tissue was deleted with the brain extraction tool from eddy current-corrected diffusion MRI data. Diffusion indices, such as FA, tensors, and the first eigenvector, were calculated using the FMRIB Diffusion Toolbox. Finally, linear and non-linear registrations were conducted using the FMRIB Linear Image Registration Tool, followed by the FMRIB Nonlinear Image Registration Tool. Automated fiber tracking with the tensor deflection method was performed with 54 white matter parcels, which were prescribed based on the Johns Hopkins University Diffusion Tensor Imaging-based white-matter atlas. The FA value at each stepping point (stepping width: 0.5 mm) along each fiber was calculated by interpolation using volume data for the center points of the nearest eight voxels around the stepping point. The terminate criteria were FA <0.25 and flip angle > 45°. Fiber tracking procedures were performed using MATLAB R2015b for Windows (MathWorks, Natick, MA, USA).

### Data Analysis

Functional image preprocessing was performed using CONN software (http://web.mit.edu/swg/software.htm). Briefly, after excluding the first 10 images to ensure signal equilibrium, functional images were corrected for head motion and temporal differences. A subject was excluded if any translation or rotation parameters in this subject's dataset exceeded ± 2.5 mm and/or ± 2.5°. The corrected functional images were first co-registered to each subject's T1 images without re-slicing. T1 images were then normalized to Montreal Neurological Institute (MNI) space, which generated a transformed matrix from native space to MNI space. Functional images were then transformed to MNI space using this matrix and resampled at 3 × 3 × 3 mm^3^. Outlier detection was performed on the normalized images. Finally, all images were smoothed with a 6 mm full width at half maximum Gaussian kernel.

An Anatomical Automatic Labeling cortical and sub-cortical atlas was used to segment the whole brain into 116 anatomical regions of interest (ROIs). For each subject, each ROI time series was extracted as the average time series across all voxels within that region. To remove spurious sources of variance, all ROI time series underwent the following steps: (1) linear detrending, (2) regressing out the six head motion parameters and their first-level derivative, the averaged cerebrospinal fluid and white matter signals, and the scrubbing signal from the time series, and (3) 0.01–0.1 Hz band-pass filtering. All of these steps were accomplished using CONN software. Finally, Pearson correlation coefficients were calculated between each pair of preprocessed ROI time series, and a temporal correlation matrix was generated for each subject. We analyzed changes in the functional connectivity of these ROIs between the two groups before and after treatment. Functional connectivity with significant group × time interactions in the two groups was investigated.

DTI data preprocessing was performed using FSL package. To compare between-group differences in white matter integrity of the uncinate fasciculus in both the right and left hemispheres, four seed regions (two seeds for each hemisphere) that represented the amygdala and MFG were defined as volumetric regions based on the anatomical automatic labeling atlas that is available within the FSL package (https://fsl.fmrib.ox.ac.uk/fsldownloads_registration). These seeds were selected to anchor the endpoint of tracks that constituted the uncinate fasciculus. For each hemisphere and each subject/visit, the uncinate fasciculus mean summary values (i.e., FA, mean diffusivity, axial diffusivity, and radial diffusivity) were extracted using TrackVis software (http://www.trackvis.org/). Finally, two-sample *t*-tests were performed to investigate white matter integrity of the uncinate fasciculus within the two groups. Significant changes in FA at baseline were correlated with neuropsychological data to investigate whether individual differences in white matter integrity were associated with the extent of depression. We reanalyzed the brain images using the false discovery rate (FDR) for multiple comparison correction.

### Statistical Analysis

Demographic and clinical characteristics were analyzed for between-group differences using two-sample *t*-tests for continuous variables and the χ^2^ test for categorical variables. The total time of voluntary mindfulness practice was summed by daily homework practice time (in hour). Repeated-measures analysis of variance (ANOVA) was used to analyze differences in clinical measures, with group (MBCT+TAU vs. TAU) and time (Pre vs. Post) as factors. Pearson correlation analyses were performed to assess correlations between significant functional connectivity findings and the clinical variables, including HAMD scores and HAMA scores, in each group separately. The tests were two-tailed. Values of *p* < 0.05 were considered statistically significant.

## Results

### Demographic Results

A total of 60 patients were recruited and randomly assigned to the MBCT+TAU group (*n* = 30; age [mean ± SD] = 62.33 ± 7.26 years) or TAU group (*n* = 30; age [mean ± SD] = 61.96 ± 5.56 years). Table 1 shows the baseline characteristics and demographics of these patients. In the randomized treatment phase, complete data were available from 30 participants (30/30, 100%) in the MBCT+TAU group. Of the 30 participants in the TAU group, 25 participants (25/30, 83.33%) had two-session MR scanning and follow-up data, four withdrew at 8-week follow-up, and one dropped out at 3 months (Figure 1). No significant differences were found between the two groups in age (*t* = 0.285, *p* = 0.776), sex ratio (χ^2^ = 0.373, *p* = 0.761), Body Mass Index (*t* = −1.773, *p* = 0.082), age at onset of depression (*t* = 0.220, *p* = 0.827), duration of depression (*t* = −0.196, *p* = 0.846), or number of episodes (*t* = −0.643, *p* = 0.523).

**Table 1:** Demographic and clinical characteristics of the participants.

	**MBCT+TAU group (*n* = 30)**	**TAU group (*n* = 30)**	***t*/*χ^2^***	***p***
Age (years)	67.66 ± 5.93	67.22 ± 5.78	0.285	0.776
Sex (% male)	6 (20%)	8 (26.7%)	0.373	0.761
Education (years)	13.73 ± 2.66	12.5 ± 3.08	1.658	0.103
Body mass index	22.64 ± 2.42	23.63 ± 1.83	−1.773	0.082
Onset age (years)	62.33 ± 7.26	61.96 ± 5.56	0.220	0.827
Duration (months)	48.23 ± 43.36	50.23 ± 35.41	−0.196	0.846
Number of episodes	2.36 ± 1.77	2.66 ± 1.84	−0.643	0.523
**Current type of medication**				
SSRIs	22	23	0.089	0.766
SNRIs	8	7	0.089	0.766

*The results are expressed as mean ± standard deviation. SSRIs, selective serotonin reuptake inhibitors; SNRIs, serotonin and norepinephrine reuptake inhibitors*.

### HAMD and HAMA Scores

**Figure 2:**
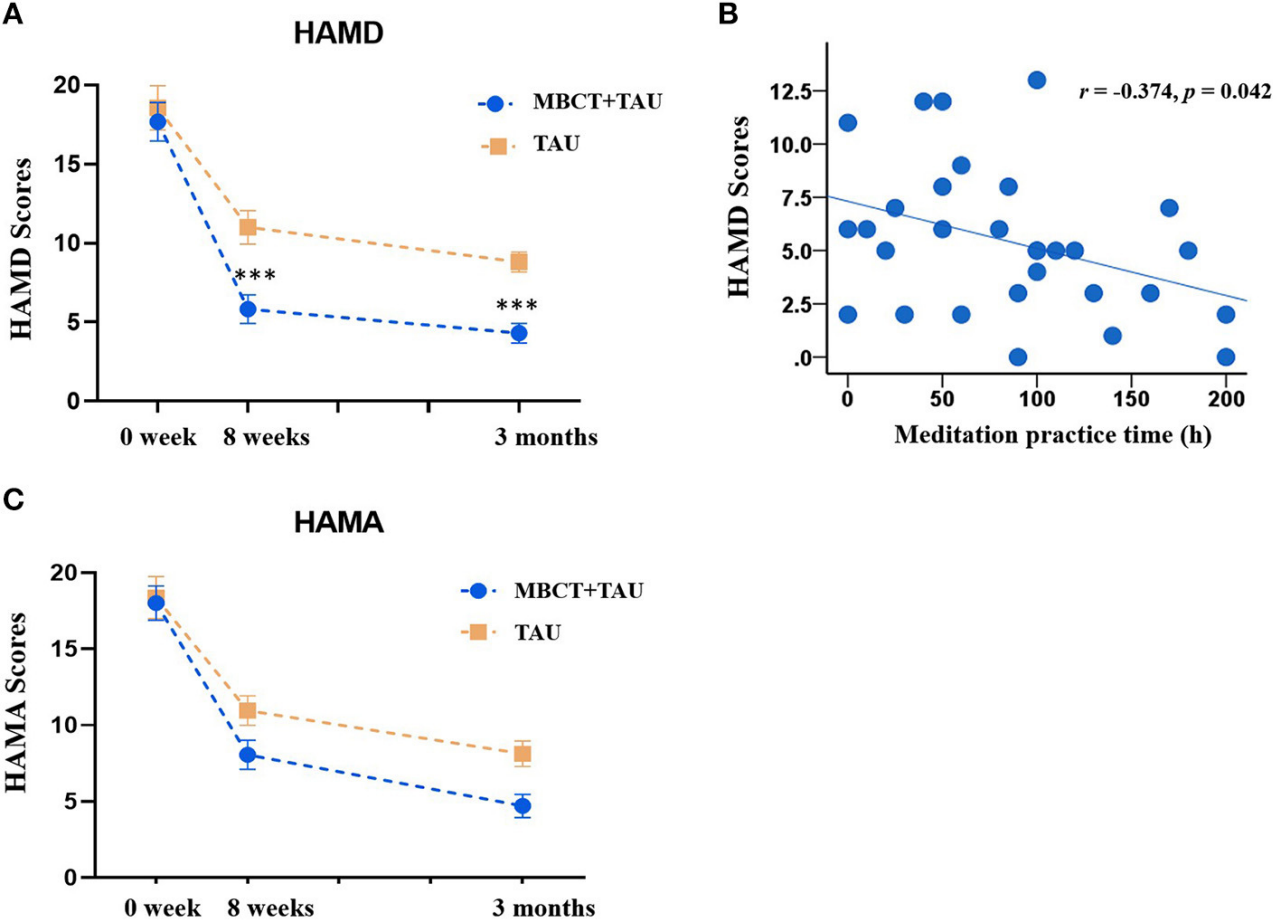
Clinical measures from baseline to endpoints in the two groups. **(A)** Changes in HAMD scores in the MBCT+TAU group and TAU group. **(B)** Correlation between meditation practice time and HAMD scores. **(C)** Changes in HAMD scores in the MBCT+TAU group and TAU group. ***p < 0.001.

For our primary outcome, changes in HAMD scores after 8 weeks of treatment and 3 months later were analyzed. For patients with adjunctive MBCT treatment, HAMD scores decreased from baseline (17.67 ± 6.68) to 8-week follow-up (5.83 ± 4.92). In the TAU group, mean HAMD scores were 18.56 ± 7.00) and 11.00 ± 5.29 at baseline and 8-week follow-up, respectively. There were no significant differences between the two groups in HAMD at baseline (*p* = 0.631). The repeated-measures ANOVA revealed main effects of time [*F*_(2,106)_ = 98.48, *p* < 0.001) and group [*F*_(1,53)_ = 12.43, *p* = 0.001] and a significant time × group interaction [*F*_(2, 106)_ = 3.38, *p* = 0.038; Figure 2A]. We found evidence of a greater reduction of depressive symptoms both after 8 weeks of treatment (*p* < 0.001) and at the end of the 3-month follow-up (*p* < 0.001), as measured by the HAMD in the MBCT+TAU group compared with the TAU group.

We also analyzed correlations between the time of voluntary mindfulness practice and improvements in depressive symptoms after treatment. Figure 2B shows that the time of voluntary mindfulness practice in the MBCT+TAU group was significantly negatively correlated with HAMD total scores at the end of treatment (*r* = −0.374, *p* = 0.042).

The repeated-measures ANOVA of HAMA scores showed main effects of time [*F*_(2,106)_ = 110.77, *p* < 0.001] and group [*F*_(1,53)_ = 4.18, *p* = 0.046] but no time × group interaction [*F*_(2,106)_ = 1.99, *p* = 0.142; Figure 2C]. Patients in both groups exhibited reductions of anxiety symptoms after treatment.

### Resting-State Functional Connectivity

**Figure 3:**
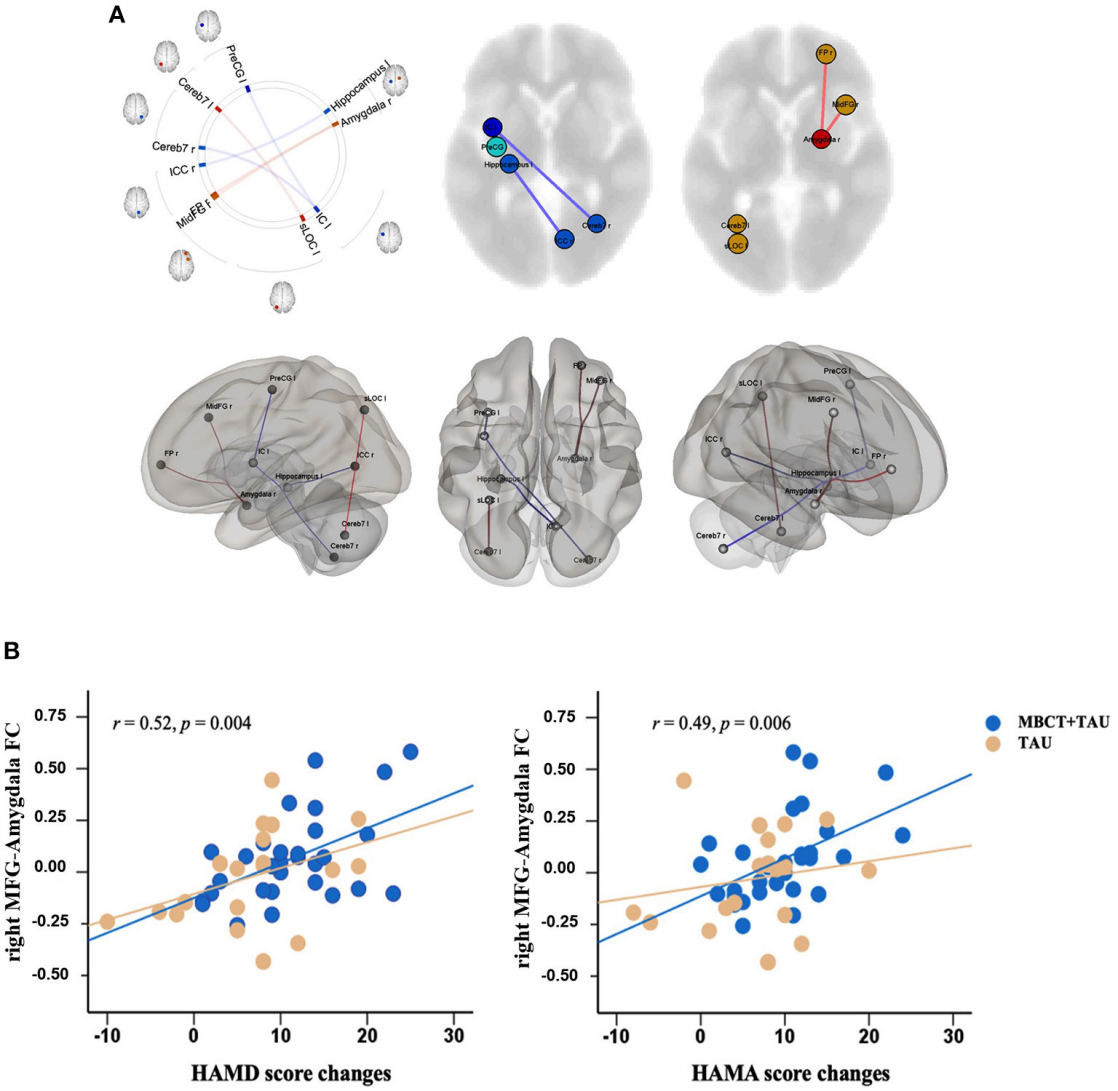
Differences in changes in resting-state functional connectivity and correlations. **(A)** Difference in changes in resting-state functional connectivity in the two groups. **(B)** Correlation between resting-state functional connectivity and clinical measures.

We found that after 8 weeks of treatment, functional connectivity of the right MFG-right amygdala, right amygdala-right frontal pole, and left supraoccipital lateral cortex-left cerebellum VII significantly increased in the MBCT+TAU group compared with the TAU group. The degree of functional connectivity of the left insula-left precentral gyrus, left insula-right cerebellum VII, and left hippocampus-right intracalcarine cortex significantly decreased (Figure 3A). Correlation analysis was used to explore the relationship between changes in functional connectivity directly and improvements in symptoms. The results showed that changes in functional connectivity of the right MFG-right amygdala in the MBCT+TAU group significantly positively correlated with changes in HAMD scores (*r* = 0.52, *p* = 0.004 after FDR corrected) and changes in HAMA scores (*r* = 0.49, *p* = 0.006 after FDR corrected), whereas no significant correlations were found in the TAU group (Figure 3B).

### Diffusion Tensor Imaging Measures

**Figure 4:**
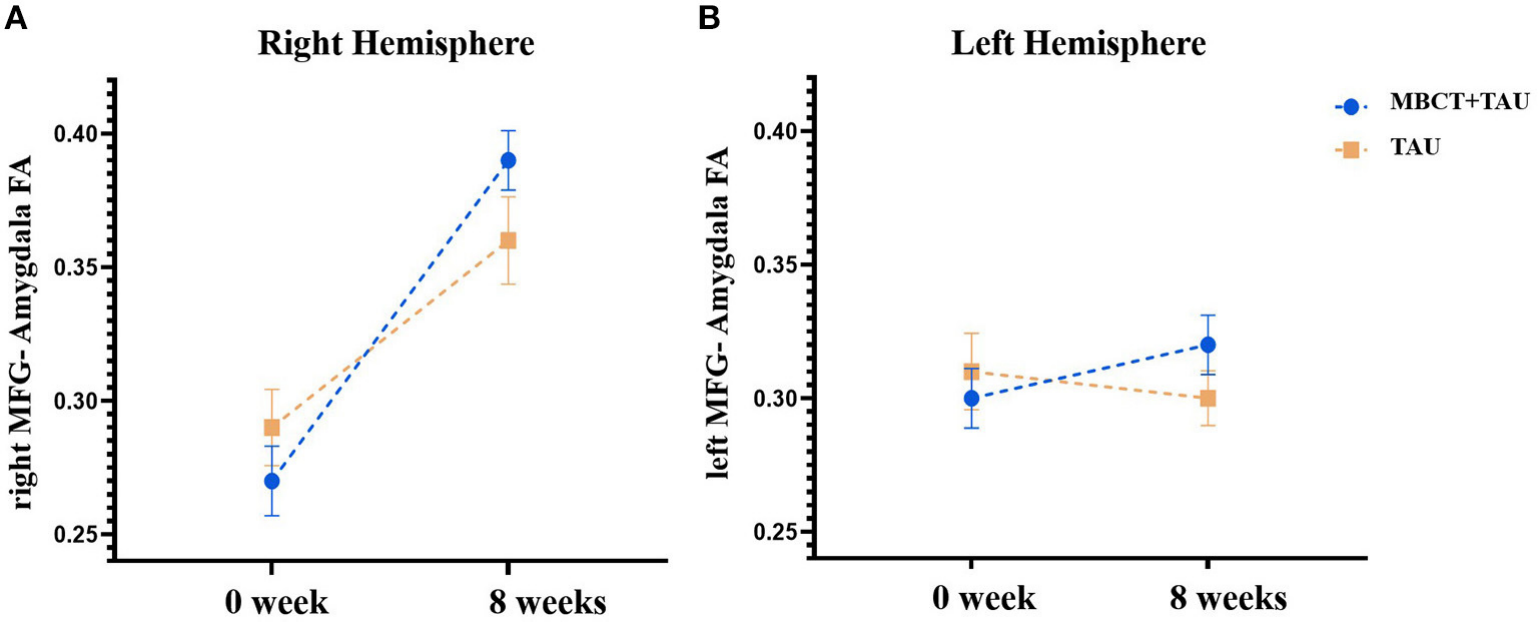
Fractional anisotropy of the MFG-amygdala before and after therapy in the two groups. **(A)** Fractional anisotropy of the right MFG-amygdala before and after therapy in the two groups. **(B)** Fractional anisotropy of the left MFG-amygdala before and after therapy in the two groups.

One participant in each group was excluded because of poor image quality for the DTI analysis. Figure 4 shows FA of the MFG-amygdala before and after therapy in the two groups. The repeated-measures ANOVA showed significant time × group [*F*_(1,51)_ = 4.829, *p* = 0.033] and time × hemisphere [*F*_(1,51)_ = 27.524, *p* < 0.001] interactions and significant main effects of time [*F*_(1,51)_ = 34.757, *p* < 0.001] and hemisphere [*F*_(1,51)_ = 5.800, *p* = 0.02] but no hemisphere × group [*F*_(1,51)_ = 0.029, *p* = 0.866] or time × hemisphere region × group [*F*_(1,51)_ = 0.335, *p* = 0.565] interaction and no main effect of group [*F*_(1,54)_ = 6.30, *p* = 0.015]. Considering the significant time × group interaction, we performed a simple-effect analysis. The results showed that FA of the MFG-amygdala in the MBCT+TAU group increased more than in the TAU group after 8 weeks of treatment, especially in the right hemisphere. All the results were corrected using FDR.

## Discussion

The present study investigated MBCT as an adjunctive treatment to TAU to reduce depression symptoms in patients with LLD and its underlying neuroimaging mechanisms. We found that 8 weeks of adjunctive MBCT with TAU resulted in superior improvement in depressive symptoms in patients with LLD. We also found that MBCT increased functional and structural connectivity between the right MFG and right amygdala, and the increase in brain connectivity positively correlated with improvements in clinical symptoms.

Depressive symptoms significantly decreased after 8 weeks of MBCT, which is consistent with previous studies (35–38). A previous study reported that patients who received MBCT experienced the significant relief of depressive symptoms, whereas the TAU group did not exhibit a significant reduction of depressive symptoms (36). An 8-week course of MBCT was more effective than sertraline monotherapy in patients with acute depression (37). Patients with LDD often experience a greater rate of anxiety symptoms, which hampers recovery (35, 39). Our findings indicated that MBCT alleviated both anxiety and depressive symptoms, achieving better outcomes than drug monotherapy. At the 3-month follow-up, a persistent decrease in depressive symptoms was observed, and HAMD scores were significantly lower in the MBCT+TAU group compared with the TAU group. These results indicate that MBCT maintained a long-term therapeutic effect in elderly individuals with LLD. We also found a positive correlation between the duration of mindfulness practice and mental health at the 3-month follow-up, which was consistent with a previous study (40). Therefore, MBCT appears to be a viable treatment option for aging patients with depression, especially individuals who suffer from undesirable side effects that are caused by pharmacotherapy.

To clarify the mechanism of MBCT, we explored changes in brain communication that were induced by MBCT using fMRI. Changes in functional connectivity of the right MFG-right amygdala in the MBCT+TAU group and improvements in HAMD scores and HAMA scores were significantly positively correlated. These results were partially consistent with previous studies that investigated neuro-mechanisms in depression patients, which also found abnormalities in connectivity in the amygdala, frontal region, insula, and hippocampus (41, 42). The amygdala is considered to be involved in affective modulation (43, 44) and has been implicated in LLD pathophysiology (45). A previous study found that responses of the amygdala were modulated by subjective experience and may reflect variations in the ability to regulate mood (46). In the present study, the increase in functional and structural connectivity between the right MFG and right amygdala could reflect or underlie alterations of the involvement of brain areas in conscious and cognitively controlled emotion processing in LLD patients after MBCT. Furthermore, functional and microstructural changes in the MFG have been shown to play a crucial role in depression (47–49). The increase in FA of the MFG correlated with a decrease in depressive symptoms (50). Our results were similar to a recent study (51) that found that patients with anxious depression exhibited lower functional connectivity between the right centromedial/laterobasal amygdala and right MFG relative to patients with non-anxious depression (51). Other studies showed that improvements in mood positively correlated with an increase in amygdala-MFG connectivity strength (52). These results indicate that MBCT may strengthen brain communication between the right MFG and right amygdala and further alleviate both anxiety and depression symptoms in LLD patients.

During 8 weeks of MBCT, meditation appeared to play an important role in improving symptoms. The time of voluntary mindfulness practice significantly negatively correlated with the severity of depressive symptoms. A previous study showed that meditation altered brain structure and neuronal plasticity (53). Cortical thickness and the activity of several specific brain regions increased during meditation, such as the hippocampus, whereas a decrease in activity was found in the amygdala (54–57). Stronger functional connectivity was found between the posterior cingulate, dorsal anterior cingulate, and prefrontal cortices was observed in experienced meditators (58, 59). Future studies are needed to explore changes in brain function and structure in patients with LLD during meditation.

Limitations of the present study should be noted. The most obvious limitation was the relatively small sample size, which may have influenced statistical power. Additionally, although we attempted to control for antidepressant use, the inclusion of patients who used medication may have confounded our findings. Furthermore, the absence of a health education control to evaluate the placebo effect of MBCT was also a limitation. Large-sample, intervention-controlled, randomized trials should be conducted to investigate the effects and underlying neural mechanisms of MBCT in patients with LDD.

## Conclusion

In conclusion, the present study provided efficacy outcomes of adjunctive MBCT with antidepressants in LLD patients. Our data indicate that changes in right MFG-amygdala connectivity were related to improvements in clinical symptoms of LLD after MBCT.

## Data Availability Statement

The original contributions presented in the study are included in the article/supplementary material, further inquiries can be directed to the corresponding authors.

## Ethics Statement

The studies involving human participants were reviewed and approved by Ethics Committee of Peking University Sixth Hospital. The patients/participants provided their written informed consent to participate in this study.

## Author Contributions

HL, XS, LLu, JD, and WY contributed to the study design. HL, XZ, WS, JD, SS, and MS performed the search and collected the data. HL, LLi, XL, and YB performed the statistical analysis. QW, LLiu, JD, and WY drafted the manuscript. All authors reviewed the results and approved the final version of the paper.

## Funding

This work was supported in part by the National Natural Science Foundation of China (nos. 82171524, 31800897, 82171477, 81821092, and 81761128036), National Key Research and Development Program of China (no. 2019YFA0706200), Capital Characteristic Project (no. Z161100000516129), and Postdoctoral Fellowship of Peking-Tsinghua Center for Life Sciences (LLiu).

## Conflict of Interest

The authors declare that the research was conducted in the absence of any commercial or financial relationships that could be construed as a potential conflict of interest.

## Publisher's Note

All claims expressed in this article are solely those of the authors and do not necessarily represent those of their affiliated organizations, or those of the publisher, the editors and the reviewers. Any product that may be evaluated in this article, or claim that may be made by its manufacturer, is not guaranteed or endorsed by the publisher.

## References

[1] Kessler RC Aguilar-Gaxiola S Alonso J Chatterji S Lee S Ormel J. The global burden of mental disorders: an update from the WHO World Mental Health (WMH) surveys. Epidemiol Psichiatr Soc. (2009) 18:23. 10.1017/s1121189x00001421 19378696 PMC3039289

[2] Alexopoulos GS. Depression in the elderly. Lancet. (2005) 365:1961–70. 10.1016/s0140-6736(05)66665-2 15936426

[3] Han LKM Aghajani M Clark SL Chan RF Hattab MW Shabalin AA. Epigenetic aging in major depressive disorder. Am J Psychiatry. (2018) 175:774–82. 10.1176/appi.ajp.2018.17060595 29656664 PMC6094380

[4] WHO. World Health Organization Depression Fact Sheet No. 369. World Health Organization (2012).

[5] Nyer M Doorley J Durham K Yeung AS Freeman MP Mischoulon D. What is the role of alternative treatments in late-life depression? Psychiatr Clin North Am. (2013) 36:577–96. 10.1016/j.psc.2013.08.012 24229658

[6] Bottino CM Barcelos-Ferreira R Ribeiz SR. Treatment of depression in older adults. Curr Psychiatry Rep. (2012) 14:289–97. 10.1007/s11920-012-0281-z 22627999

[7] DeRubeis RJ Siegle GJ Hollon SD. Cognitive therapy versus medication for depression: treatment outcomes and neural mechanisms. Nat Rev Neurosci. (2008) 9:788–96. 10.1038/nrn2345 18784657 PMC2748674

[8] Segal ZV Williams M Teasdale J. Mindfulness-Based Cognitive Therapy for Depression. 2nd ed. New York, NY: Guilford Press (2012)

[9] Mathew KL Whitford HS Kenny MA Denson LA. The long-term effects of mindfulness-based cognitive therapy as a relapse prevention treatment for major depressive disorder. Behav Cogn Psychother. (2010) 561–76. 10.1017/S135246581000010X 20374671

[10] Kuyken W Warren FC Taylor RS Whalley B Crane C Bondolfi G. Efficacy of mindfulness-based cognitive therapy in prevention of depressive relapse: an individual patient data meta-analysis from randomized trials. JAMA Psychiatry. (2016) 73:565–74. 10.1001/jamapsychiatry.2016.0076 27119968 PMC6640038

[11] Williams JMG Kuyken W. Mindfulness-based cognitive therapy: a promising new approach to preventing depressive relapse. Br J Psychiatry. (2012) 200:359–60. 10.1192/bjp.bp.111.104745 22550328

[12] Teasdale JD Segal ZV Williams JM Ridgeway VA Soulsby JM Lau MA. Prevention of relapse/recurrence in major depression by mindfulness-based cognitive therapy. J Consult Clin Psychol. (2000) 68:615–23. 10.1037//0022-006x.68.4.615 10965637

[13] Torres-Platas SG Escobar S Belliveau C Wu J Sasi N Fotso J. Mindfulness-based cognitive therapy intervention for the treatment of late-life depression and anxiety symptoms in primary care: a randomized controlled trial. Psychother Psychosom. (2019) 88:254–6. 10.1159/000501214 31288245

[14] Dikaios E Escobar S Nassim M Su CL Torres-Platas SG Rej S. Continuation sessions of mindfulness-based cognitive therapy (MBCT-C) vs. treatment as usual in late-life depression and anxiety: an open-label extension study. Int J Geriatr Psychiatry. (2020) 35:1228–32. 10.1002/gps.5360 32525235

[15] Kaiser RH Andrews-Hanna JR Wager TD Pizzagalli DA. Large-scale network dysfunction in major depressive disorder: a meta-analysis of resting-state functional connectivity. JAMA Psychiatry. (2015) 72:603–11. 10.1001/jamapsychiatry.2015.0071 25785575 PMC4456260

[16] Williams LM. Precision psychiatry: a neural circuit taxonomy for depression and anxiety. Lancet Psychiatry. (2016) 3:472–80. 10.1016/S2215-0366(15)00579-9 27150382 PMC4922884

[17] Schmaal L Hibar D Sämann PG Hall G Baune B Jahanshad N. Cortical abnormalities in adults and adolescents with major depression based on brain scans from 20 cohorts worldwide in the ENIGMA Major Depressive Disorder Working Group. Mol Psychiatry. (2017) 22:900–9. 10.1038/mp.2016.60 27137745 PMC5444023

[18] Videbech P Ravnkilde B. Hippocampal volume and depression: a meta-analysis of MRI studies. Am J Psychiatry. (2004) 161:1957–66. 10.1176/appi.ajp.161.11.1957 15514393

[19] Treadway MT Waskom ML Dillon DG Holmes AJ Park MTM Chakravarty MM. Illness progression, recent stress, and morphometry of hippocampal subfields and medial prefrontal cortex in major depression. Biol Psychiatry. (2015) 77:285–94. 10.1016/j.biopsych.2014.06.018 25109665 PMC4277904

[20] Sheline YI Gado MH Kraemer HC. Untreated depression and hippocampal volume loss. Am J Psychiatry. (2003) 160:1516–8. 10.1176/appi.ajp.160.8.1516 12900317

[21] Serra-Blasco M Portella MJ Gomez-Anson B de Diego-Adelino J Vives-Gilabert Y Puigdemont D. Effects of illness duration and treatment resistance on grey matter abnormalities in major depression. Br J Psychiatry. (2013) 202:434–40. 10.1192/bjp.bp.112.116228 23620451

[22] Lacerda AL Nicoletti MA Brambilla P Sassi RB Mallinger AG Frank E. Anatomical MRI study of basal ganglia in major depressive disorder. Psychiatry Res Neuroimaging. (2003) 124:129–40. 10.1016/S0925-4927(03)00123-9 14623065

[23] Leaver AM Yang H Siddarth P Vlasova RM Krause B St Cyr N. Resilience and amygdala function in older healthy and depressed adults. J Affect Disord. (2018) 237:27–34. 10.1016/j.jad.2018.04.109 29754022 PMC5995579

[24] Li H Lin X Liu L Su S Zhu X Zheng Y. Disruption of the structural and functional connectivity of the frontoparietal network underlies symptomatic anxiety in late-life depression. Neuroimage Clin. (2020) 28:102398. 10.1016/j.nicl.2020.102398 32919365 PMC7491145

[25] Lifshitz M Sacchet MD Huntenburg JM Thiery T Fan Y Gärtner M. Mindfulness-based therapy regulates brain connectivity in major depression. Psychother Psychosom. (2019) 88:375–7. 10.1159/000501170 31509824

[26] Fox KC Dixon ML Nijeboer S Girn M Floman JL Lifshitz M. Functional neuroanatomy of meditation: a review and meta-analysis of 78 functional neuroimaging investigations. Neurosci Biobehav Rev. (2016) 65:208–28. 10.1016/j.neubiorev.2016.03.021 27032724

[27] Fox KC Nijeboer S Dixon ML Floman JL Ellamil M Rumak SP. Is meditation associated with altered brain structure? A systematic review and meta-analysis of morphometric neuroimaging in meditation practitioners. Neurosci Biobehav Rev. (2014) 43:48–73. 10.1016/j.neubiorev.2014.03.016 24705269

[28] Kral TRA Schuyler BS Mumford JA Rosenkranz MA Lutz A Davidson RJ. Impact of short- and long-term mindfulness meditation training on amygdala reactivity to emotional stimuli. Neuroimage. (2018) 181:301–13. 10.1016/j.neuroimage.2018.07.013 29990584 PMC6671286

[29] Zhang A Leow A Ajilore O Lamar M Yang S Joseph J. Quantitative tract-specific measures of uncinate and cingulum in major depression using diffusion tensor imaging. Neuropsychopharmacology. (2012) 37:959–67. 10.1038/npp.2011.279 22089322 PMC3280650

[30] Cullen KR Klimes-Dougan B Muetzel R Mueller BA Camchong J Houri A. Altered white matter microstructure in adolescents with major depression: a preliminary study. J Am Acad Child Adolesc Psychiatry. (2010) 49:173–83.e1. 10.1097/00004583-201002000-00011 20215939 PMC2909686

[31] Ma X Liu J Liu T Ma L Wang W Shi S. Altered resting-state functional activity in medication-naive patients with first-episode major depression disorder vs. healthy control: a quantitative meta-analysis. Front Behav Neurosci. (2019) 13:89. 10.3389/fnbeh.2019.00089 31133831 PMC6524692

[32] Peters AT Burkhouse K Feldhaus CC Langenecker SA Jacobs RH. Aberrant resting-state functional connectivity in limbic and cognitive control networks relates to depressive rumination and mindfulness: a pilot study among adolescents with a history of depression. J Affect Disord. (2016) 200:178–81. 10.1016/j.jad.2016.03.059 27136416

[33] Lin X Li W Dong G Wang Q Sun H Shi J. Characteristics of multimodal brain connectomics in patients with schizophrenia and the unaffected first-degree relatives. Front Cell Dev Biol. (2021) 9:631864. 10.3389/fcell.2021.631864 33718367 PMC7947240

[34] Lin X Deng J Dong G Li S Wu P Sun H. Effects of chronic pharmacological treatment on functional brain network connectivity in patients with schizophrenia. Psychiatry Res. (2021) 295:113338. 10.1016/j.psychres.2020.113338 32768152

[35] Foulk MA Ingersoll-Dayton B Kavanagh J Robinson E Kales HC. Mindfulness-based cognitive therapy with older adults: an exploratory study. J Gerontol Soc Work. (2014) 57:498–520. 10.1080/01634372.2013.869787 24329497

[36] Barnhofer T Crane C Hargus E Amarasinghe M Winder R Williams JM. Mindfulness-based cognitive therapy as a treatment for chronic depression: A preliminary study. Behav Res Ther. (2009) 47:366–73. 10.1016/j.brat.2009.01.019 19249017 PMC2866254

[37] Eisendrath SJ Gillung E Delucchi K Mathalon DH Yang TT Satre DD. A preliminary study: efficacy of mindfulness-based cognitive therapy versus sertraline as first-line treatments for major depressive disorder. Mindfulness. (2015) 6:475–82. 10.1007/s12671-014-0280-8 26085853 PMC4465797

[38] Eisendrath SJ Gillung E Delucchi KL Segal ZV Nelson JC McInnes LA. A randomized controlled trial of mindfulness-based cognitive therapy for treatment-resistant depression. Psychother Psychosom. (2016) 85:99–110. 10.1159/000442260 26808973 PMC4756643

[39] Fiske A Wetherell JL Gatz M. Depression in older adults. Annu Rev Clin Psychol. (2009) 5:363–89. 10.1146/annurev.clinpsy.032408.153621 19327033 PMC2852580

[40] Chiesa A Castagner V Andrisano C Serretti A Mandelli L Porcelli S. Mindfulness-based cognitive therapy vs. psycho-education for patients with major depression who did not achieve remission following antidepressant treatment. Psychiatry Res. (2015) 226:474–83. 10.1016/j.psychres.2015.02.003 25744325

[41] Pannekoek JN Van Der Werff S Meens PH van den Bulk BG Jolles DD Veer IM. Aberrant resting-state functional connectivity in limbic and salience networks in treatment-naive clinically depressed adolescents. J Child Psychol Psychiatry. (2014) 55:1317–27. 10.1111/jcpp.12266 24828372

[42] Luking KR Repovs G Belden AC Gaffrey MS Botteron KN Luby JL. Functional connectivity of the amygdala in early-childhood-onset depression. J Am Acad Child Adolesc Psychiatry. (2011) 50:1027–41. e3. 10.1016/j.jaac.2011.07.019 21961777 PMC3185293

[43] Gallagher M Chiba AA. The amygdala and emotion. Curr Opin Neurobiol. (1996) 6:221–7. 10.1016/s0959-4388(96)80076-6 8725964

[44] Murray EA. The amygdala, reward and emotion. Trends Cogn Sci. (2007) 11:489–97. 10.1016/j.tics.2007.08.013 17988930

[45] Burke J McQuoid DR Payne ME Steffens DC Krishnan RR Taylor WD. Amygdala volume in late-life depression: relationship with age of onset. Am J Geriatr Psychiatry. (2011) 19:771–6. 10.1097/JGP.0b013e318211069a 21873832 PMC3164525

[46] Dyck M Loughead J Kellermann T Boers F Gur RC Mathiak K. Cognitive versus automatic mechanisms of mood induction differentially activate left and right amygdala. Neuroimage. (2011) 54:2503–13. 10.1016/j.neuroimage.2010.10.013 20946960

[47] Yang Q Huang X Hong N Yu X. White matter microstructural abnormalities in late-life depression. Int Psychogeriatr. (2007) 19:757–66. 10.1017/S1041610207004875 17346365

[48] Taylor WD MacFall JR Payne ME McQuoid DR Provenzale JM Steffens DC. Late-life depression and microstructural abnormalities in dorsolateral prefrontal cortex white matter. Am J Psychiatry. (2004) 161:1293–6. 10.1176/appi.ajp.161.7.1293 15229065

[49] Dutta A McKie S Deakin JF. Resting state networks in major depressive disorder. Psychiatry Res. (2014) 224:139–51. 10.1016/j.pscychresns.2014.10.003 25456520

[50] Peng H Zheng H Li L Liu J Zhang Y Shan B. High-frequency rTMS treatment increases white matter FA in the left middle frontal gyrus in young patients with treatment-resistant depression. J Affect Disord. (2012) 136:249–57. 10.1016/j.jad.2011.12.006 22217432

[51] Qiao J Tao S Wang X Shi J Chen Y Tian S. Brain functional abnormalities in the amygdala subregions is associated with anxious depression. J Affect Disord. (2020) 276:653–9. 10.1016/j.jad.2020.06.077 32871697

[52] Bershad AK Preller KH Lee R Keedy S Wren-Jarvis J Bremmer MP. Preliminary report on the effects of a low dose of LSD on resting-state amygdala functional connectivity. Biol Psychiatry Cogn Neurosci Neuroimaging. (2020) 5:461–7. 10.1016/j.bpsc.2019.12.007 32033922 PMC7150630

[53] Afonso RF Kraft I Aratanha MA Kozasa EH. Neural correlates of meditation: a review of structural and functional MRI studies. Front Biosci. (2020) 12:92–115. 10.2741/s542 32114450

[54] Luders E Thompson PM Kurth F Hong JY Phillips OR Wang Y. Global and regional alterations of hippocampal anatomy in long-term meditation practitioners. Hum Brain Mapp. (2013) 34:3369–75. 10.1002/hbm.22153 22815233 PMC4084509

[55] Lazar SW Bush G Gollub RL Fricchione GL Khalsa G Benson H. Functional brain mapping of the relaxation response and meditation. Neuroreport. (2000) 11:1581–5. 10.1097/00001756-200005150-00041 10841380

[56] Davanger S Ellingsen O Holen A Hugdahl K. Meditation-specific prefrontal cortical activation during acem meditation: an fMRI study. Percept Mot Skills. (2010) 111:291–306. 10.2466/02.04.22.PMS.111.4.291-306 21058608

[57] Doll A Holzel BK Mulej Bratec S Boucard CC Xie X Wohlschlager AM. Mindful attention to breath regulates emotions via increased amygdala-prefrontal cortex connectivity. Neuroimage. (2016) 134:305–13. 10.1016/j.neuroimage.2016.03.041 27033686

[58] Brewer JA Worhunsky PD Gray JR Tang YY Weber J Kober H. Meditation experience is associated with differences in default mode network activity and connectivity. Proc Natl Acad Sci USA. (2011) 108:20254–9. 10.1073/pnas.1112029108 22114193 PMC3250176

[59] Berkovich-Ohana A Harel M Hahamy A Arieli A Malach R. Alterations in task-induced activity and resting-state fluctuations in visual and DMN areas revealed in long-term meditators. Neuroimage. (2016) 135:125–34. 10.1016/j.neuroimage.2016.04.024 27109713

